# Predictive factors for the medical hospitalisation of patients who visited the emergency department with suicide attempt

**DOI:** 10.1186/s12888-021-03089-2

**Published:** 2021-02-06

**Authors:** Hye Jin Kim, Duk Hee Lee

**Affiliations:** 1 Department of Emergency Medicine, Sanggye Paik Hospital, Inje University College of Medicine, Seoul, Republic of Korea; 2 Department of Emergency Medicine, Ewha Womans University, Anyangchoenro 1071 Yangcheon-gu, Seoul, Republic of Korea

**Keywords:** Emergency department, Hospitalisation, Length of stay, Suicide attempt

## Abstract

**Background:**

Suicide is a significant public health problem. Individuals are estimated to make up to 20 suicide attempts before suicide. The emergency department (ED) is the first location where individuals are brought after a suicide attempt. This study investigated the factors related to delays in the medical hospitalisation of patients who attempted suicide and aimed to provide criteria for hospitalisation decisions by physicians.

**Methods:**

This study included patients who had deliberately self-harmed (age ≥ 19 years) and presented at the EDs of two tertiary teaching hospitals between March 2017 and April 2020. Those for whom relevant demographic and clinical information were unavailable and those admitted to the psychiatric wards were excluded.

**Results:**

This study included 414 patients in the hospitalisation group and 1346 in the discharged group. The mean patient age was 50.3 ± 20.0 years and 40.7 ± 17.0 years in the hospitalised and discharged groups (*p* < 0.001), respectively. The mean ED length of stay (LOS) was 4.2 ± 12.3 and 11.4 ± 18.8 h in the hospitalised and discharged groups, respectively. In the hospitalised group, the odds ratio and confidence interval for aged 35 ~ 64 (2.222, 1.343–3.678), aged over 65 (2.788, 1.416–5.492), sex -male (2.041, 1.302–3.119), and consciousness (1.840, 1.253–2.466). The Risk-Rescue Ratio Scale (RRRS) was (1.298, 1.255–1.343). A receiver operating characteristics analysis of RRRS for the decision to hospitalise patients who attempted suicide showed a cut-off value of 42, with sensitivity, specificity, and area under the curve being 85.7, 85.5%, and 0.924, respectively.

**Conclusion:**

The level of consciousness and the RRRS of patients who attempted suicide can be the factors to decide medical hospitalisation and reduce ED LOS and crowding.

**Supplementary Information:**

The online version contains supplementary material available at 10.1186/s12888-021-03089-2.

## Background

Suicide is a significant public health problem. South Korea has a high suicide rate [1], which was ranked first among the member countries of the Organization for Economic Cooperation and Development (OECD) during 2003–2017 and second since 2018 [2]. South Korea has seen a rapid increase in the suicide rate, which has tripled since the nineties [3]. Moreover, individuals are estimated to make up to 20 suicide attempts before suicide [4]. The emergency department (ED) is the first contact point providing management for patients who have attempted suicide when primary care institutions or outpatient clinics are inaccessible [5]. Patients with physical injuries or altered mental status, besides psychiatric issues, inevitably tend to visit the ED [6]. The hospitals and EDs are readily accessible through South Korea’s universal healthcare system [7].

In 2018, according to the statistics issued by the Korea Suicide Prevention Centre, based on information from the death certificates in Statistics Korea and the Emergency Department Information System, there were 13,670 deaths by suicide and 33,451 patients who attempted suicide [8].

Most of the patients who attempt suicide receive medical care through the ED, and both domestic and international research on these individuals is frequently based on injury-monitoring data or medical records from the ED and ED patients [9–12]. The patients who require only psychiatric care are typically either discharged after a psychiatric consultation or admitted to a psychiatric ward, while those with an altered mental status, in need of intensive care or surgery, due to physical injuries, may require medical hospitalisation. The hospitalisation decision is based on the clinician’s judgement. Suicidal patients may be discharged or hospitalized after being assessed according to available guidelines. However, these guidelines are not universally accepted [13]. During the management of patients who have attempted suicide, the decision for hospitalisation is as crucial as the acute stabilisation of their concurrent injuries [14]. There are some studies on the variables associated with only psychiatric hospitalisation [15, 16] as well as both medical and psychiatric hospitalisation [17]. Currently, there are no reference materials or quantified guidelines for medical hospitalisation of suicide attempt patients in South Korea emergency department.

The risk-rescue ratio scale (RRRS) was validated for its psychometric properties [13] and has been used to determine the lethality of suicide attempts [14]. For instance, Oh et al. showed that deliberate self-poisoning was included in the high-risk/low-rescue group [15]. Kim et al. analyzed several psychologic scales used to determine the hospitalisation need of suicidal patients in the ED and reported that RRRS was the most useful tool for predicting hospitalisation in the ED setting [16]. A few previous studies have suggested that suicide mortality can be lowered if individuals at risk of suicide are effectively identified and appropriately treated. In the busy ED, physicians do not use scoring systems due to the limited time and high patient volumes [12]. Therefore, a guideline to aid early decision-making for hospitalisation of suicide attempt patients would help to avoid ED crowding. Furthermore, ED crowding is a significant patient safety concern associated with poor quality of care [17], and a significant positive relationship between ED crowding and patient mortality has been reported [18, 19]. Henneman showed that both hospitalised and discharged patients who stayed in the ED for 6 h were associated with ED crowding [20]. There is an ongoing investigation into ED crowding and ED capacity in South Korea [21]. In the ED, emergency medicine physicians, depending on the severity of the patient’s condition, evaluate and treat those who attempt suicide and decide upon their subsequent care. Assessment of the suicide attempt patients by the first or second-year residents with lack of experience or inexperienced physicians results in patient delays in the ED. Furthermore, there are cases that the patients’ conditions may worsen due to lack of proper assessment and management in the ED.

This study investigated the factors related to delays in medical or surgical hospitalisation of suicide attempt patients and aimed to provide criteria to assist the physicians’ rapid decision making. This can helpshorten the decision time for hospitalisation and the ED length of stay (LOS), hence ensure proper treatment of the patients.

## Methods

### Study population, data collection, and variables

This retrospective cohort study included adult patients who attempted suicide (≥19 years) and visited the EDs at two tertiary teaching hospitals from March 2017 to April 2020. These patients had deliberately self-harmed with overdoses (drugs, pesticides, herbicides, chemicals), and injuries (fall, drowning, hanging, cutting, collision). At each study centre, there were two coordinators, certified by the South Korean Psychologic Counselling Association. The research team comprised a psychologist, an emergency medicine specialist, and two coordinators. The coordinators had been educated regularly by the Life love crisis response team of Korea Ministry of Health and Welfare. The research team surveyed all patients who visited the ED after a suicide attempt. The initial assessment forms were designed under the supervision of psychiatrists. The patient data were collected prospectively, and the data were reviewed retrospectively by the researchers.

Each hospital was located in a metropolitan area and provided healthcare services to a population of approximately 700,000 individuals, with an annual average of 60,000 patient visits at each hospital. The hospitalisation process was as follows. If a patient needed surgery, then the surgery department hospitalised the patient. If a patient was in a state of shock or had an altered mentality (such as drug overdose, carbo-monoxide poisoning, hanging, etc.), the emergency medicine department hospitalised the patient. All the patients or their guardians were consulted by the on-duty psychiatrist. If the patient did not require hospitalisation, the psychiatric department decided on psychiatric admission or discharge. Coordinators also met with the patients and their guardians. The case management team held regular conferences every two weeks to discuss data biases and improvements. All these details are uploaded onto the data collecting site of the Ministry of Health and Welfare, the project host.

The assessment, designed in consultation with psychiatrists, collected demographic and clinical information, including age, sex, vital signs (systolic blood pressure, diastolic blood pressure, and heart rate), time from suicide attempt to ED visit, location where the suicide was attempted, patients’ marital status, religion, employment status, income level (with reference to the average monthly income of Korean workers, classified on the basis of 1.5 and 2.5 million KRW), education level (with reference to the mandatory education in South Korea), cohabitant, consciousness (alert–verbal response–pain response–unresponsiveness), method of suicide attempt, alcohol ingestion before the suicidal attempt, request for help, history of suicidal attempts, history of psychiatric care, psychiatric drug use, history of psychiatric admission, and suicidal attempt plan.

Information on ED outcomes (intensive care unit [ICU] admission, general ward [GW] admission, discharge, and death), presumptive psychiatric diagnosis (depression, psychiatric disease other than depression, and no intervention or inability to diagnose), and physical status at the time of visit to the ED (chronic disease, acute disease, and physically healthy) was collected.

The RRRS was calculated using the formula: [Risk rating/(Risk rating + Rescue rating)] × 100 (risk and rescue ratings are presented in Appendix).

The data were collected, and the RRRS was determined by a senior emergency medicine resident under the supervision of an emergency medicine specialist and the on-duty psychiatrists. The psychiatric diagnoses were made by psychiatric residents by using the criteria specified in the Diagnostic and Statistical Manual of Mental Disorders-IV (American Psychiatric Association, 2000).

### Statistical analysis

The patients were assigned to two groups: the hospitalisation group and the discharge group to analyse the factors which effect on the duration of ED length of stay. The hospitalisation group included all patients admitted to the ICU and GW. The mean with standard deviation and median with 25th and 75th percentiles were expressed as the continuous variables. The number of patients and percentage were expressed as the noncontinuous variables. For items requiring statistical verification, an independent T-test was used for the continuous variables and Chi-squared tests were used for the non-continuous variables. A *p*-value < 0.05 was considered statistically significant. We selected all variables that proved to be statistically significant (*p* < 0.05) from the univariate analysis and performed multivariate logistic regression analysis using backward stepwise selection (likelihood ratio); variables with *p* < 0.05 are listed in the table. Statistical analyses were conducted using the Statistical Package for the Social Sciences (version 21) for Windows (International Business Machines Corporation, Armonk, NY, USA), and 95% confidence intervals with statistically significant *p*-values were reported.

## Results

**Fig. 1:**
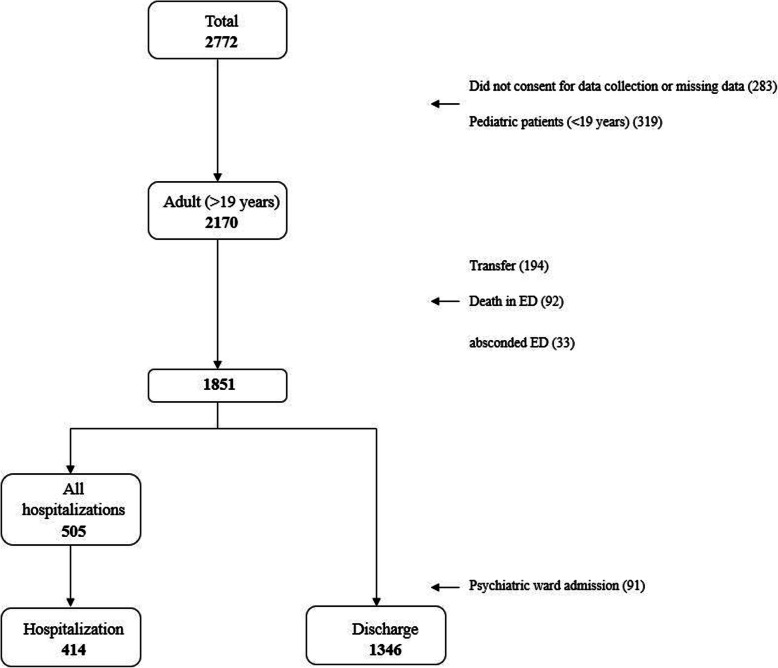
Patient selection flow

During the study period, 2772 individuals attempted suicide, out of which 319 were paediatric patients and 602 were patients who were either transferred or who had died in the ED were excluded. Furthermore, out of the remaining 1851 patients, 91 patients admitted to a psychiatric ward were also excluded. After the exclusions, a total of 414 and 1346 patients were included in the hospitalised and discharged groups, respectively (Fig. 1).

### Comparison of general characteristics of suicide attempted patients between hospitalised and discharged groups

Table 1 presents the general characteristics of patients who attempted suicide and visited the ED during the study period. In the hospitalised and discharged groups, the mean age was 50.3 ± 20.0 and 40.7 ± 17.0, respectively, and the number of males was 183 (44.2%) and 420 (31.2%), respectively (*p* < 0.0001). In the age groups of < 34 years old, 35–65 years old, and over 65 years, there were significant differences were observed (*p* < 0.0001). There were more patients in the hospitalised group, in the 35–65-year age group (47.1%) and the over 65 years age group (27.1%) than in the discharged group. There was also a significant intergroup difference in the marital status (*p* < 0.001). Single status was more prevalent (45.7%) in the discharged group compared with the hospitalised group (35.2%). In the discharged group 48.3% had the highest income (> 2.5 million KRW/month) compared with 38.1% in the hospitalised group, while 28.7% in the discharged group had the lowest income (< 1.5 million KRW/month) compared with 43.7% in hospitalised group. Educational status, religion, cohabitant, insurance, time from suicide attempt to ED visit, and location of suicide attempt did not show significant between-group differences. The ED LOS significantly differed between the hospitalised and the discharged groups (11.4 ± 18.8 h vs 4.2 ± 12.3 h; *p* < 0.002). ED LOS subgroup (~ 2 h, 2–6 h, over 6-h) showed that longer ED LOS was associated with a greater possibility of hospitalisation (*p* < 0.001).

**Table 1:** Comparison of the general characteristics of patients who attempted suicide between the hospitalized and discharged groups

	Discharge (*n* = 1346)	Hospitalization (*n* = 414)	Total (*n* = 1760)	*p*-value
Age (years, mean ± SD)	40.7 ± 17.0	50.3 ± 20.0	42.7 ± 18.1	0.000
Sex, n (%)				0.000
Male	420 (31.2)	183 (44.2)	603 (33.9)	
Female	926 (68.8)	231 (55.8)	1163 (66.1)	
Marital status, n (%)				0.001
Single	615 (45.7)	146 (35.2)	761 (43.6)	
Married	572 (42.5)	201 (48.5)	773 (43.7)	
Separated	116 (8.6)	38 (9.3)	154 (8.8)	
Widowed	43 (3.2)	29 (6.9)	72 (3.9)	
Education status, no. (%)				0.305
Elementary school and lower	75 (5.6)	37 (9.0)	112 (6.3)	
Middle school	61 (4.5)	21 (5.0)	82 (4.6)	
High school	605 (44.9)	179 (43.2)	784 (44.6)	
University and higher	605 (44.9)	177 (42.8)	782 (44.5)	
Religion, n (%)				0.409
Yes	392 (29.1)	137 (33.1)	529 (30.0)	
No	954 (70.9)	277 (66.9)	1231 (70.0)	
Employment, n (%)				0.021
Yes	594 (44.1)	152 (36.6)	746 (42.6)	
No	752 (55.9)	262 (63.4)	1014 (57.4)	
Cohabitant, n (%)				0.186
Yes	937 (69.6)	305 (73.6)	1242 (70.4)	
No	409 (30.4)	109 (26.4)	518 (29.6)	
Health status, n (%)				0.000
Healthy	993 (73.8)	257 (62.1)	1250 (71.4)	
Acute disease	24 (1.8)	11 (2.6)	35 (2.0)	
Chronic disease without disability	125 (9.3)	48 (11.7)	173 (9.8)	
Chronic disease with disability	204 (15.1)	98 (23.5)	299 (16.9)	
Income, n (%)				0.003
< 1.5 million KRW/month	386 (28.7)	181 (43.7)	567 (31.4)	
1.5–2.5	310 (23.0)	76 (18.3)	386 (22.2)	
> 2.5	650 (48.3)	157 (38.1)	807 (46.4)	
Insurance, n (%)				0.057
National health insurance	1176 (87.4)	373 (90.8)	1549 (88.1)	
Medicaid beneficiary	141 (10.5)	33 (8.0)	174 (10.0)	
Self-pay (uninsured)	29 (2.1)	8 (1.3)	37 (1.9)	
Time from attempting to ED visit (hours)	7.1 ± 33.1	5.9 ± 21.4	6.81 ± 31.0	0.518
ED Length of stay (hours, mean ± SD)	4.2 ± 12.3	11.4 ± 18.8	5.4 ± 13.7	0.002
Location of suicide attempt, n (%)				0.778
Home	1170 (86.9)	350 (84.6)	1520 (86.4)	
School	1 (0.1)	0 (0.0)	1 (0.1)	
Workplace	10 (0.8)	3 (0.8)	13 (0.8)	
Lodging	22 (1.6)	17 (4.0)	39 (2.2)	
Car	16 (1.2)	2 (0.5)	18 (1.1)	
Group living facility	1 (0.1)	2 (0.5)	3 (0.2)	
Healthcare facility	7 (0.5)	1 (0.3)	8 (0.4)	
Commercial facility	23 (1.7)	10 (2.5)	33 (1.9)	
River	51 (3.8)	12 (3.0)	63 (3.7)	
Public facility	10 (0.8)	7 (1.8)	17 (1.0)	
Others	35 (2.4)	10 (2.1)	45 (2.3)	

*ED LOS* emergency department length of stay

### Comparison of suicidal attempt-related patient characteristics

Table 2 showed the differences between the study groups with regards to the characteristics of the patients who attempted suicide. There was a significant between-group difference in the degree of mental alertness at the time of ED visit (*p* < 0.001). Patients who did not request help had a higher rate of hospitalisation (*p* < 0.026). Patients with a previous suicidal attempt had a higher rate of discharge. Previous suicidal attempts were 2.5 ± 2.9 in the discharged group and 1.7 ± 1.4 in the hospitalised group (*p* < 0.009). Alcohol ingestion before self-harm was 50.4% in the discharged group and 42.8% in the hospitalised group (*p* < 0.011) There were no significant intergroup differences in the histories of psychiatric ward admissions, current psychiatric medication use, and psychiatric tentative diagnoses. The motivation for the suicidal attempt (*p* < 0.001) and the method of the suicidal attempt (*p* < 0.001) differed significantly between the groups.

**Table 2:** Comparison of the characteristics of the patients who attempted suicide who visited the ED

	Discharge	Hospitalization	Total	*p*-value
Consciousness, n (%)				0.001
Alert	1004 (74.6)	189 (45.7)	1193 (68.3)	
Verbal response	249 (18.5)	95 (22.9)	344 (19.5)	
Pain response	93 (6.9)	107 (25.8)	200 (10.9)	
Unresponsiveness	0 (0.0)	23 (5.6)	23 (1.3)	
Vital signs				
Systolic blood pressure(mmHg)	122.6 ± 21.8	118.5 ± 28.8	121.0 ± 25.4	0.007
Diastolic blood pressure(mmHg)	74.5 ± 15.1	70.2 ± 18.0	73.3 ± 17.1	0.001
Pulse rate(beat/min)	91.5 ± 19.6	91.1 ± 23.4	90.9 ± 21.8	0.421
Respiratory rate(/min)	19.9 ± 2.0	20.0 ± 2.9	19.8 ± 2.9	0.297
Body temperature (°C)	36.6 ± 1.1	36.2 ± 2.7	56.3 ± 3.0	0.035
Request for help				0.026
Yes	701 (52.1)	188 (45.5)	889 (50.7)	
No	645 (47.9)	226 (54.5)	871 (49.3)	
Previous suicidal attempt				0.001
Yes	568 (42.2)	118 (28.5)	686 (39.3)	
No	778 (57.8)	296 (71.5)	1074 (60.7)	
Number of previous suicidal attempts (*n* = 686) (mean ± SD)	2.5 ± 2.9	1.7 ± 1.4	2.3 ± 2.6	0.009
Past psychiatric consultation				0.017
Yes	758 (56.3)	206 (49.8)	964.0 (55.0)	
No	588 (43.7)	208 (50.2)	796.0 (45.0)	
History of psychiatric admission				0.078
Yes	253 (18.8)	58 (14.0)	311 (17.9)	
No	1093 (81.2)	356 (86.0)	1449 (82.1)	
Current psychiatric medication use				0.544
Yes	810 (60.2)	240 (58.0)	1050 (59.7)	
No	536 (39.8)	174 (42.0)	710 (40.3)	
Alcohol ingestion before suicidal attempt				0.011
Yes	678 (50.4)	177 (42.8)	855 (48.9)	
No	668 (49.6)	237 (57.2)	905 (51.1)	
Planed suicidal attempt				
Yes	109 (8.1)	72 (17.5)	181 (9.9)	0.001
No	1237 (91.9)	342 (82.5)	1579 (90.1)	
Motivation of the suicidal attempt				0.001
Psychiatric	1147 (48.8)	303 (52.3)	1450 (49.5)	
Interpersonal	442 (18.8)	84 (14.5)	526 (18.0)	
Job-related	122 (5.2)	26 (4.5)	148 (5.1)	
Economic	131 (5.6)	36 (6.2)	167 (5.8)	
Illness-related	129 (5.5)	46 (8.0)	175 (6.0)	
Death of family member or pet	42 (1.8)	11 (1.9)	53 (1.8)	
Legal problem	19 (0.8)	2 (0.3)	21 (0.7)	
Loneliness	25 (1.1)	6 (1.1)	31 (1.1)	
Fighting or punishment	233 (9.9)	54 (9.4)	287 (9.8)	
Other traumatic event	54 (2.5)	11 (1.9)	65 (2.2)	
Method of the suicidal attempt				0.001
Medication poisoning	709 (52.7)	219 (53.0)	929 (52.7)	
Pesticides and herbicides	28 (2.1)	32 (7.8)	61 (3.3)	
Gas poisoning	50 (3.7)	9 (2.2)	59 (3.4)	
Chemical exposure	28 (2.1)	17 (4.1)	45 (2.6)	
Hanging	67 (5.0)	23 (5.6)	90 (5.2)	
Drowning	20 (1.5)	4 (1.0)	24 (1.4)	
Cutting and piercing	326 (24.2)	70 (17.0)	396 (22.7)	
Fall/jumping from a height	20 (1.5)	18 (4.4)	38 (2.1)	
Collision/burns	4 (0.3)	1 (0.2)	5 (0.3)	
Others	93 (6.9)	19 (4.6)	112 (6.4)	
Psychiatric tentative diagnosis in ED				0.430
Depressive disorder	1118 (83.1)	343 (82.8)	1461 (83.0)	
Bipolar disorder	66 (4.9)	16 (3.8)	82 (4.7)	
Adjustment disorder	63 (4.7)	21 (5.0)	84 (4.7)	
Schizophrenia and schizotypal and delusional disorders	57 (4.2)	21 (5.0)	77 (4.4)	
Substance-use disorder	15 (1.1)	5 (1.3)	20 (1.2)	
Anxiety disorder	11 (0.9)	0 (0.0)	11 (0.7)	
Personality disorder	9 (0.7)	3 (0.8)	13 (0.7)	
Organic mental disorder	3 (0.2)	3 (0.8)	6 (0.3)	
Somatoform disorders	3 (0.2)	2 (0.4)	4 (0.2)	
Disorders of psychological development	1 (0.1)	0 (0.0)	1 (0.1)	
Risk-Rescue Ratio Scale score	36.7 ± 8.3	50.4 ± 8.8	39.4 ± 9.8	0.001

### Multiple logistic regression analysis for prediction of hospitalisation

**Fig. 2:**
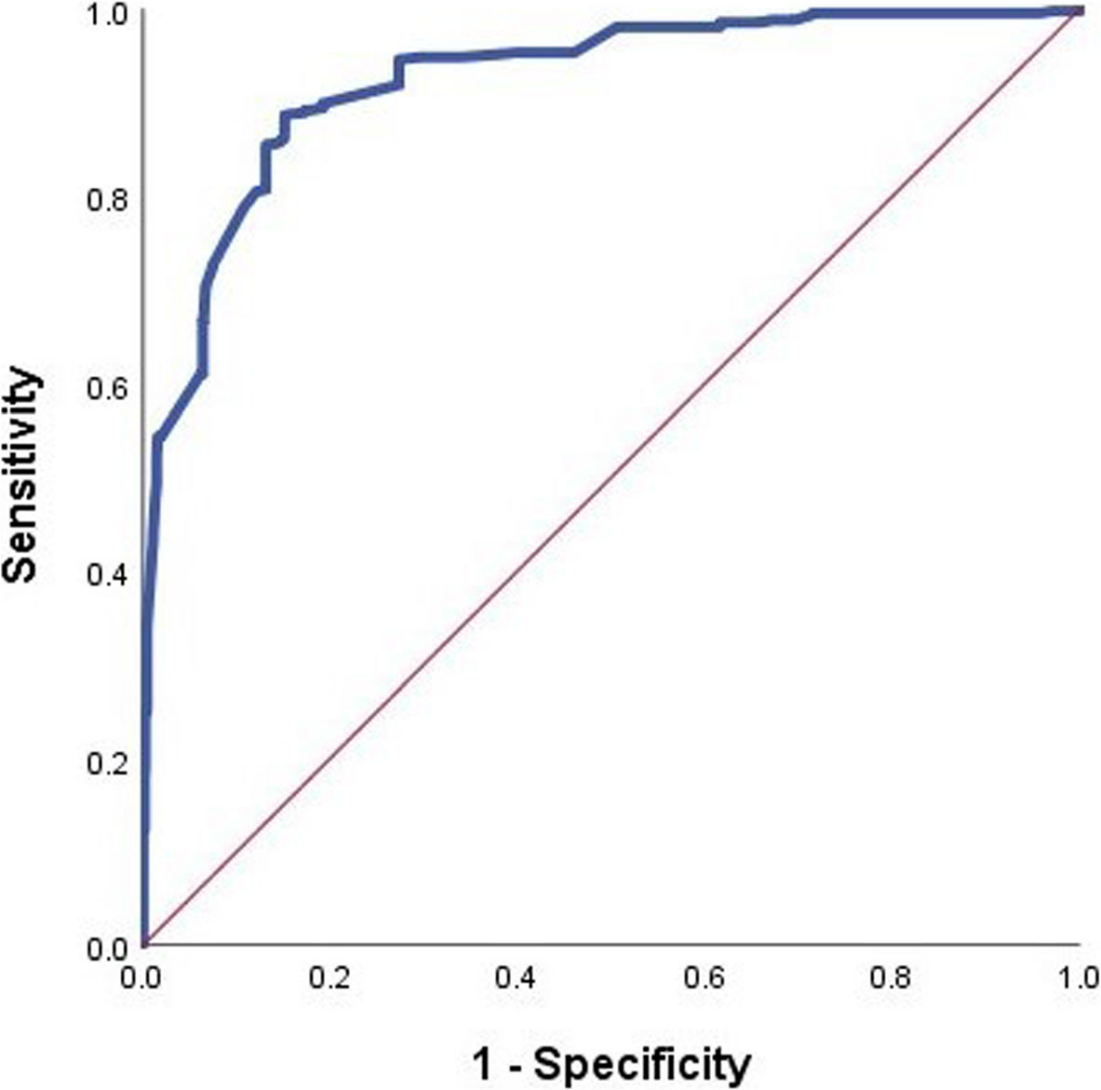
The receiver operating characteristics (ROC) analysis of the Risk-Rescue Ratio Scale (RRRS) in predicting the hospitalization of patients who attempted suicide. A cutoff value of 42.9, with 85.7% sensitivity, 85.5% specificity and an area under the curve of 0.924 [95% CI, 0.909–0.938]

In the hospitalised group, the logistic regression showed statistically significant differences between the age subgroup and ED LOS subgroup. The odds ratio (OR) and confidence interval (CI) of the age subgroups 35–65 and over 65 years old were 2.222 (1.343–3.678) and 2.788 (1.416–5.492), respectively, while the ED LOS subgroups 2–6 and over 6-h were 1.674 (0.998–2.808) and 1.771 (1.017–3.083), respectively. The OR and CI of the study variables for sex (male) and consciousness (alert vs. verbal response, pain responses, and unresponsiveness) were 2.041(1.302–3.119) and 1.840(1.253–2.466), respectively. The RRRS was 1.298 (1.255–1.343) (Table 3). The OR of the receiver operating characteristics analysis of RRRS for the association with the hospitalisation decision reguarding suicidal patients showed a cut-off value of 42.9, with an 85.7% sensitivity, 85.5% specificity, and an area under the curve of 0.924 (Fig. 2).

**Table 3:** Early decision factors identified in the hospitalized group by multivariate logistic regression analysis

Variables	Univariate					Multivariate				
	OR	(95% CI)			*p*-value	OR	(95% CI)			*p*-value
Age subgroup (Years)										
(≤34)	1.000	Reference			–	1.000	Reference			–
(35–64)	1.722	(1.330	–	2.228)	0.000	2.222	(1.343	–	3.678)	0.002
(65≤)	5.014	(3.639	–	6.908)	0.000	2.788	(1.416	–	5.492)	0.003
Sex, male	1.750	(1.402	–	2.284)	0.000	2.041	(1.302	–	3.119)	0.002
ED LOS (hours)										
(~ 1.5)		Reference			–	1.000	Reference			–
(1.5–6)	1.481	1.048	–	2.093	0.026	1.674	(0.998	–	2.808)	0.051
(6~)	1.933	1.333	–	2.802	0.001	1.771	(1.017	–	3.083)	0.043
Consciousness	3.499	(2.792	–	4.385)	0.000	1.840	(1.253	–	2.466)	0.000
RRRS	1.275	(1.245	–	1.305)	0.000	1.298	(1.255	–	1.343)	0.000

## Discussion

The findings of this study demonstrated that the methods of suicide attempt had an effect on the need for hospitalisation. Among the OECD countries, South Korea has the highest suicide rate, which is two times higher than that in the United States [22]. While suicide ranks as the 13th leading cause of life lost with 800,000 deaths worldwide annually [23], it is the 5th leading cause of death in South Korea [24]. The ED is an important point-of-care in the healthcare process for patients who have attempted suicide. In this study, drug overdose was the most common method of suicide (52.4% of the total patients), and had similar rates in both the hospitalised and discharged groups. Walker et al. had reported drug overdose to be a strong predictor of ICU admission [25] while Kim et al. classified stabbing, hanging, drowning, and jumping off a height as clinically serious, lethal methods, and severe depression, psychological disturbance, and repetitive suicidal ideation as factors that indicated high medical severity [26]. This study excluded patients who died while in the ED or within 24 h of hospitalisation. Those patients represented less than 10% of all suicide attempted patients.

Those who were discharged from the hospital in this study were younger by approximately 10 years and were more likely to be female (68.8%). Age may be a factor in that the younger are discharged because they are healthier and recover better. Gender as a risk factor for suicide has been studied extensively. Female attempted non-fatal suicide behaviour [27, 28]. Several studies have highlighted the concept of non-suicidal self-harm (NSSI) and have distinguished between suicidal attempts and NSSI [29, 30]. It has been reported that NSSI attempts consists of patients that are younger and more frequently female and that these attempts are less fatal with more frequent use of cutting methods [31]. Data in this study included all patients with deliberate self-harm without questioning the authenticity of the suicide attempts. Further research is needed on whether there are many NSSI patients in the discharged group.

Studies have shown associations between alcohol use disorder and suicidal behaviour [32, 33]. Salles et al. reported that an alcohol use disorder was not associated with hospitalisation for inpatient psychiatric care, whereas depression was a clearly associated factor [34]. This present study also showed that alcohol use was not associated with hospitalisation; rather, alcohol use before suicidal behavior was higher in the discharged group. The higher rate of alcohol use rate in the discharged group may show that the alcohol causes non-serious impulsive suicide behaviour.

Mortin J reported that in the elderly with suicide intentions alcohol use disorder showed a strong association with hospital-treated suicide [35]. In Baltic countries, there was a high suicide rate, but due to their restrictive alcohol policy, there was a lower annual male suicide rate during 1986–80 [36].

South Korea ranks first in suicide rate and twenty-three in alcohol consumption [37]. Thus, the association between suicide rate and alcohol consumption may not be clear. However, this study only investigated whether the patients consumed alcohol and not whether the patients were diagnosed with an alcohol use disorder. The amount of alcohol consumed and the blood alcohol concentration were not ascertained. These aspects need to be evaluated in future research.

One in eight patients who visit the ED is a psychiatric patient, contributing to ED overcrowding [38, 39]. The ED LOS is associated with increased ED crowding. The walk-out rate was 0.34 patients/hour when the ED LOS was less than 6 h and 0.77 patients per hour when the patients’ ED LOS was more than 6 h [20]. In this study, the average ED LOS in the hospitalized group was 11.4 ± 18.8 h vs 4.2 ± 12.3 h in the discharged group, which significantly contributed to the ED crowding. The overall ED LOS of patients with psychiatric emergencies and suicidal/self-harm patients had a median duration of 2.4 h, which showed that the duration had been maintained at a similar value for 3 years, with local differences [40].

In this study, the hospitalised group had a significantly longer ED LOS than the discharged group, with an average duration above 6 h. Besides the time for assessment and treatment, the time to make hospitalisation decisions contributed to the long ED LOS. In both hospitals where this study was conducted, the hospitalisation decision for patients who attempted suicide via drug overdose, hanging, and drowning was made by department of emergency medicines. South Korea does not have a dedicated toxicology department at hospitals, leaving the hospitalisation decision to the department of emergency medicine or internal medicine physicians. Therefore, the ED LOS is extended when the patients cannot be admitted to a psychiatric ward due to physical injuries and is even longer with ED crowding.

In this study, the ORs for hospitalisation based on mental status at presentation were 2.027 for verbal response, 6.200 for pain response, and 39.931 for unresponsiveness. The OR for consciousness was 1.840, indicating that to avoid ED crowding and improve the ED occupancy ratio, emergency physicians should not delay the hospitalisation decision when the patients were not alert. Jo et al. reported that ED crowding was associated with a higher mortality rate in critically ill patients [41]. In addition to the level of alertness, the socioeconomic status can affect the hospitalisation decision. Even in alert patients, a report estimates that approximately 25% of patients could have been discharged if they had social support, and that clinical severity alone does not determine the need for hospitalisation [42]. In this study, consultancy care was provided to the caregivers when hospitalized patients had altered mental status, and for unconscious patients who received a psychiatric consultation after they regained consciousness. All hospitalised patients were transferred or discharged after the psychiatric follow-up consultation.

Our study is different from previous reports as it analyses ED LOS, evaluate factors that affect ED LOS, and emphasizes the need to prevent delays in the ED to ensure adequate in-hospital treatment. The RRRS had high sensitivity and specificity for the hospitalization decision of deliberated self-harm in this study. In the RRRS, the absence of loss of consciousness, confusion, and coma are assigned 1, 2, and 3 points, respectively. It does not have a large effect on the total score. Based on a regression analysis, the level of consciousness should be considered as a single factor for the hospitalisation decision. The RRRS and mental status are objective indicators that provide meaningful guidelines to aid hospitalization decisions. Weiland et al. reported that emergency physicians were uniformly confident deciding to shift patients at risk of suicide or self-harm to the inpatient department [43]. This study could provide guidance to emergency physician in South Korea or countries with similar situations.

The planned suicide attempt was twice as frequently in the hospitalisation group than in the discharge group (OR 1.728). A previous report indicated that planned suicidal attempts have severe medical consequences [44]. For patients with a planned suicidal attempt, a psychiatric follow-up after initial care is important.

In this study, the history of psychiatric care or current psychiatric medication usage did not differ significantly between the study groups. This may suggest that suicidal behaviour was present regardless of psychiatric treatment, or that psychiatric treatment was inadequate to prevent suicidal attempts. Harada et al. reported that females tended to be over-represented in the psychiatric consultation group, and males in the non-consultation group. Poisoning by prescription drugs was used more frequently as a method of suicide in the consultation group. Moreover, the prevalence of adult personality disorders and schizophrenia and related disorders were higher in the consultation group than in the non-consultation group [45].

Shepard et al. reported that based on the reported numbers alone, the national cost of suicides and suicidal attempts in the United States in 2013 was $58.4 billion. After adjustment for under-reporting, the cost increased to a total of $93.5 billion, which was 2.1–2.8 times that reported in previous studies [46]. In South Korea, statistics on suicidal attempts are only available through ED data. Furthermore, the cost of medical care and related indirect costs have not been investigated. Considering the high prevalence of suicide in South Korea, if the direct and indirect costs of ED use due to deliberate self-harm and by under-reporting of suicide attempts are studied, the national cost of suicide is likely to be high. Further research is needed to assess the national costs associated with suicide.

This study had several limitations. One limitationis the failure of many patients to participate in the data collection or patient exclusion due to missing data. Moreover, the study was conducted at tertiary teaching hospitals in metropolitan areas and may not be representative of ED situations in rural areas. Further research should include multiple levels of hospitals nationwide to identify other issues in the medical hospitalisation process of patients who attempt suicide.

In this study, patients in the ICU and GW were grouped in the hospitalised group and compared with those in the ED discharged group. Since close observation is available in the ICU in both the study hospitals, patients who attempted suicide by drug overdose, hanging, and drowning were hospitalised and admitted to the ICU if they had a high risk of suicide re-attempt even if their physical injury was treatable in the GW. Therefore, the hospitalisation rate for the ICU and GW was difficult to determine, and it was more appropriate to include both as the total hospitalised group. Furthermore, continuous psychiatric consultation is not provided during hospitalisation and is only provided following discharge. Another limitation is that bed availability at the time of hospitalisation was not assessed. It may be possible that an early hospitalisation decision was made, but a bed was not immediately available. However, this may not add a significant bias as patients are typically transferred to another hospital in case of unavailability soon after the hospitalisation decision.

This study was also limited by the absence of long-term findings such as the results of before and after admission to the emergency room due to the inability to conduct a longitudinal study. In this study, data were collected when entering and leaving the emergency room. Although the coordinators have not changed, residents change annually. Therefore, hospitalization decisions by different doctors may vary.

This study included 2.6% of ED visits by patients who were 19 years of age or older without an existing illness in South Korea. When suicidal/self-harming patients desired medical hospitalisation due to physical injury, there is an extra barrier to the hospitalization decision for departments other than psychiatry due to the combination of the patients’ psychiatric condition, with their physical injury. The present study demonstrates that the level of consciousness and RRRS scores can be used as factors for the hospitalization in patients who attempted suicide. In future, a longitudinal study, including adolescents, will be meaningful.

## Conclusions

Suicidal patients often end up in the ED, and they frequently require medical hospitalisation rather than psychiatric admission due to their physical injury. ED overcrowding increases the mortality risk in critical patients and reduces the quality of care. When considering the medical hospitalisation of patients who attempted suicide, the level of consciousness and the RRRS can help make medical hospitalisation decisions to reduce crowding and improve patient safety.

## Supplementary Information

**Additional file 1.** Risk Rescue Ratio Scale.

## Data Availability

There are ethical restrictions on sharing a dataset because the data contain potentially identifying information. Suicide case management team can be contacted for data access via e-mail (ewha_lovelife@naver.com) or by calling 82-2-2650-5296.

## Abbreviations

ED: Emergency Department
ED LOS: Emergency department length of stay
RRRS: Risk-Rescue Rating Scale
OECD: Organization for economic cooperation and development
LOS: Length of stay
CI: Confident Interval
GW: General ward
ICU: Intensive care unit
