# Supplementary material for: Predictive factors for the medical hospitalisation of patients who visited the emergency department with suicide attempt
Source: BMC Psychiatry. 2021 Feb 6;21:79. doi: 10.1186/s12888-021-03089-2 (PMC7866662; doi:10.1186/s12888-021-03089-2)
Supplement: Supplementary file 1 — Additional file 1. Risk Rescue Ratio Scale. [file 12888_2021_3089_MOESM1_ESM.docx]

Appendix. Risk Rescue Ratio Scale

| Risk factors | Points | | |
| --- | --- | --- | --- |
|  | 1 | 2 | 3 |
| Agent used | Ingestion, cutting, stabbing | Drowning, Asphyxia, strangulation | Jumping, shooting |
| Impaired consciousness | Non in evidence | Confusion, semi-coma | Coma, deep coma |
| Lesions/toxicity | Mild | Moderate | Severe |
| Reversibility | Good, complete recovery expected | Fair, recovery expected with time | Poor, residuals expected if recovery |
| Treatment required | First aid, emergency room care | Admission, routine treatment | Intensive care, special treatment |
| Rescue factors | Points | | |
|  | 3 | 2 | 1 |
| Location | Familiar | Non-familiar, non-remote | remote |
| Person initiating rescue (In case of self-rescue, the rescue score = 5) | Key person | Professional | Passer by |
| Probability of discovery by any rescuer | High, almost certain | Uncertain discovery | Accidental discovery |
| Accessibility to rescue | Asks for help | Drops clues | Does not ask for help |
| Delay until discovery | Immediate ~ 1 hour | Less than 4 hours | Greater than 4 hour |

(Korean version of RRRS)

| **자살 위험도 및 구조 상황 평가** | | | | | | |
| --- | --- | --- | --- | --- | --- | --- |
| **(Risk-Rescue Ratio = Risk 총점 / (Risk 총점 + Rescue 총점) x 100= ( ) / ( + ) x 100 = ( ) 점** | | | | | | |
| ***스스로 구조되었을 경우에는 자동적으로 Rescue 점수가 5점으로 부여** | | | | | | |
| ****발견된 이후 치료를 받기까지 지연이 되었을 경우 Rescue 총점에서 1점 감점** | | | | | | |
| **점수** | | **1점** | **2점** | **3점** | **환자** | **총점** |
| **Risk** | **시도방법** | 음독/손목자해/찌름 | 익수/목맴/질식 | 투신/총기 발사 |  |  |
| **(자살 위험도)** | **의식손상** | 없음 | 혼란(confuse) | 혼미, 혼수(coma) |  |  |
|  | **병변의 심각성** | 경도(mild) | 중등도(moderate) | 중증(severe) |  |  |
|  | **회복가능성** | 우수, | 양호, 회복이 기대 | 불량, 회복 후에도 |  |  |
|  |  | 완전한 회복 기대 | 되나 시간이 필요 | 후유증 예상 |  |  |
|  | **필요한 처치** | 응급실 내 간단한 | 일반 병동 입원 및 일상적 치료 | 중환자실 혹은 |  |  |
|  |  | 응급 처치 |  | 특수치료(수술 등) |  |  |
| **Rescue** | **자살시도장소** | 익숙한 곳(집, 회사) | 가까운 곳 | 먼 곳, 외딴장소 |  |  |
| **(구조 가능도)** | **구조를 시작한사람** | 시도자에게 중요한 인물 | 전문가(응급구조사,의료인) | 지나가던 행인, |  |  |
|  |  |  |  | 모르는 사람 |  |  |
|  | **발견 가능성** | 거의 확실히 구조 | 발견이 불확실 | 우연히 발견됨 |  |  |
|  | **구조를 위한 접근성** | 도움을 청함 | 단서를 남김 | 도움을 청하지 않음 |  |  |
|  | **구조되기까지 시간** | 즉각, 1시간 이내 | 4시간 미만 | 4시간 이상 |  |  |
